# Neuroprotective Consequences of Postconditioning on Embolic Model of Cerebral Ischemia in Rat

**Published:** 2013-02

**Authors:** Hosseinali Rezazadeh, Mohammadamin Hoseini Kahnuee, Ali Roohbakhsh, Ali Shamsizadeh, Mohammad Reza Rahmani, Reza Bidaki, Fatemeh Amin, Bahareh Kamali, Hamid bakhshi, Mohammad Allahtavakoli

**Affiliations:** 1 Physiology-Pharmacology Research Centre, Rafsanjan University of Medical Sciences, Rafsanjan, Iran; 2 Moradi Hospital, Dept of Psychiatry, Rafsanjan University of Medical Sciences, Rafsanjan, Iran; 3 Medical Education Center, Rafsanjan University of Medical Sciences, Rafsanjan, Iran; 4 Pharmacy Research Centre, School of Pharmacy, Mashhad University of Medical Sciences, Mashhad, Iran

## Abstract

**Objective(s)**: It has been reported that ischemic postconditioning, conducted by a series of brief occlusion and release of the bilateral common carotid arteries, confers neuroprotection in permanent or transient models of stroke. However, consequences of postconditioning on embolic stroke have not yet been investigated.

***Materials and Methods: ***In the present study, rats were subjected to embolic stroke (n=30) or sham stroke (n=5). Stroke animals were divided into control (n=10) or three different patterns of postconditioning treatments (n=20). In the first pattern of postconditioning (PC10, n=10), the common carotid arteries (CCA) were occluded and reopened 10 and 30 sec, respectively for 5 cycles. Both occluding and releasing times in pattern 2 (PC30, n=5) and 3 (PC60, N=5) of postconditionings, were five cycles of 30 or 60 sec, respectively. Postconditioning was induced at 30 min following the stroke. Subsequently, cerebral blood flow (CBF) was measured from 5 min before to 60 min following to stroke induction. Infarct size, brain edema and neurological deficits and reactive oxygen species (ROS) level was measured two days later.

***Results:*** While PC10 (*P<*0.001), PC30 and PC60 (*P<*0.05) significantly decreased infarct volume, only PC10 decreased brain edema and neurological deficits (*P<*0.05). Correspondingly, PC10 prevented the hyperemia of brain at 35, 40, 50 and 60 min after the embolic stroke (*P<*0.005). No significant difference in ROS level was observed between PC10 and control group.

***Conclusion: ***Ischemic postconditioning reduces infarct volume and brain edema, decreases hyperemia following to injury and improves neurological functions after the embolic model of stroke.

## Introduction

Ischemic stroke is the third leading cause of death and the primary cause of disability in adults worldwide. Stroke is accompanied by a robust inflammatory response, glutamate mediated excitotoxicity, release of reactive oxygen species (ROS) and the initiation of apoptosis (1). The current lack of clinical treatment for acute stroke requires the exploration of new treatments that may eventually lead to a viable clinical application. One of these concepts is ischemic postconditioning (2).

 Ischemic postconditioning is defined as a series of rapid intermittent interruptions of blood flow in the early phase of reperfusion that mechanically alters the hydrodynamics of reperfusion (3). In the brain, ischemic postconditioning is conducted by a series of brief occlusion and release of the bilateral common carotid arteries (CCA) following to reperfusion(4). While little is known about its mechanisms, numerous investigators recently demonstrated that ischemic postconditioning protects brain from injury in transient (5-7) or permanent MCA occlusion models. An investigation suggested that ischemic postconditioning may not influence the early brain damage induced by focal cerebral ischemia in rats (8). Hence, further studies are required to address this controversy. 

Embolic stroke model, induced by natural old clots, is more relevant to the pathophysiological situation in patients, because the majority of ischemic injuries in humans are induced by old thrombi that originate from the heart and carotid arteries (9). The effect of postconditioning has not yet been investigated on embolic stroke. Therefore, the objective of the present study was to investigate the effects of postconditioning, conducted by the repetitive occluding and releasing of the bilateral common carotid artery (CCA), on hyperemia, infarct volume, brain edema, ROS activity and neurological outcome following embolic stroke in rats.

## Materials and Methods

*Animals and treatments*

Animals were handled in accordance with criteria outlined in the Guide for Care and Use of Laboratory Animals (NIH US publication 86-23 revised 1985; http://oacu.od.nih.gov/regs/guide/guidex.htm). Experimental protocols were approved by animal ethic committee of Rafsanjan University of Medical Sciences. A total of 35 male Wistar rats weighing 200 to 250 grams were maintained on a 12 hr light-dark cycle with food and water available ad libitum. Animals were randomly assigned to control (n=10), Sham operated (n=5) and three different patterns of postconditioning treatment (n=20) as describe below. In a separate experiment, 10 animals were assigned to control or PC10 (n=5 for each group) for ROS level measurement.

*Surgical and clot preparation*

Rats were anesthetized with halothane (5% for induction and 1.5-2% for maintenance) and subjected to embolic stroke. Body temperature was maintained normothermic (37oC) throughout. For preparation of embolus, the femoral artery of the donor rat was catheterized and blood was transferred directly into a 20 cm length of PE-50 tube and kept for 2 hr at room temperature. The clot was subsequently refrigerated for 22 hr at 4oC prior to use. A 2 cm segment of clot filled PE-50 tube was cut and clot expelled from the tube and transferred into another PE-50 modified tube with an outer diameter of 0.3 mm for injection into MCA (10). 

*CBF measurement*

Relative regional cerebral blood flow was monitored with laser doppler monitor (LD-CBF) extracranially. After the scalp was opened and the right temporalis muscle was gently separated from the bone, the right skull bone was thinned by a dental drill. For CBF measurement, the probe was attached to the bone 1 mm posterior and 5 mm mediolateral to the bregma. CBF was measured 5 min before clot injection as the baseline value, at the time of ischemia, and at 10, 20, 30, 35, 40, 50 and 60 min intervals after stroke. A minimum initial reduction of 70% in the laser doppler reading was considered a successful occlusion of the MCA perfusion territory (11). CBF values were expressed as percentages relative to baseline (100%).

*Induction of embolic stroke model*

The embolic stroke model was induced by embolizing a preformed clot into the MCA as reported formerly (12). Briefly, a longitudinal incision of 1.5 cm in length was made in the midline of the ventral cervical skin. The right common carotid artery, internal carotid artery, and external carotid artery were exposed. The distal portion of the external carotid artery was ligated and cut. The modified PE-50 tubing with the 20 mm clot was connected to a 50-µl Hamilton lock syringe, and advanced 17-19 mm in the internal carotid artery until its tip was inside of the MCA. The clot was then injected, and the catheter was removed. The wound was closed, and the animal was returned to its cage. For the sham operated animals 5 µl saline was injected into the MCA. The stroke surgeon was blind to the group of animals he was working on. The duration of surgery did not exceed 30 min in any case.

*Ischemic postconditioning*

**Figure 1:**
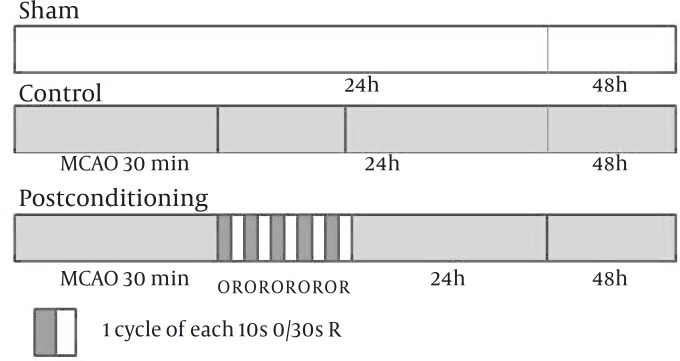
Experimental protocol used to determine the effect of Postconditioning after the embolic model middle cerebral artery occlusion (MCAO). Sham indicates sham-operated rats; Control, rats subjected to embolic stroke and followed for 48 hr; Postconditioning, rats treated with ischemic Postconditioning after 30 min of stroke induction and followed for 48 hr. Postconditioning was induced by 5 cycles of occluding and releasing common carotid arteries for 10 or 30 sec, respectively. O and R indicate Occluding and Reopening, respectively

Postconditioning was performed after 30 min of MCA occlusion (MCAo) by the clot. Postconditioning was executed by occluding bilateral common carotid arteries (CCA), by using 4-0 silk strings that already tied loosely around CCAs. To explore optimal method for the protective effects of postconditioning, we examined three different patterns of postcoditionings. In the first pattern of postconditioning (PC10, n=10), the CCAs occluding and releasing times were 10 and 30 sec, respectively and repeated for 5 cycles (Figure 1). In pattern 2 (PC30, n=5) and 3 (PC60, n=5), both occluding and releasing times were 30 or 60 sec, respectively, and repeated for 5 cycles (13). 

*Measurement of infarct volumes and Brain edema*

The quantification of infarct volume has been previously described (6). Briefly, all animals sacrificed 48 hr after the onset of MCA occlusion. The brains were removed from the skull and cut into 2-mm-thick coronal sections in a cutting block and stained with 2% 2,3, 5- triphenyltetrazolium chloride (Sigma) for 30 min at 37°C followed by overnight immersion in 10% formalin. The infarcted tissue remained unstained (white), whereas normal tissue was stained red. The infarct zone was demarcated and analyzed by Image J software (NIH Image, version 1.61). Infarct areas of all sections were added to determine the total infarct area, which was multiplied by the thickness (2 mm) of the brain sections to obtain the infarct volume. To compensate for the effect of brain edema, the corrected infarct volume was calculated as follows: corrected infarct area=measured infarct area×(1-[(ipsilateral hemisphere area-contralateral hemisphere area) /contralateral hemisphere]) (6). Brain edema was determined with the following formula: edema= (volume of right hemisphere-volume of left hemisphere)/volume of left hemisphere (12). The brain edema was expressed as percentages.

*Behavioral test*

Neurological deficits were recorded at 24 and 48 hr following to embolic stroke and determined with a modified 6-point scoring system of Bederson and coworkers (14) as follows: 0, no observable deficit; 1, forelimb flexion; 2, forelimb flexion plus decreased resistance to lateral push; 3, unidirectional circling; 4, unidirectional circling plus decreased level of consciousness; and 5, death.

*Detection of ROS generation*

Since dihydroethidium (DHE) fluorescence can be used to detect both intracellular and extracellular superoxide detection (15), the generation of pri-infarct ROS was assessed by DHE (Sigma, USA). Briefly, fresh brains were quickly frozen at -80oC until being sectioned by the cryosection (E1100325, SLEE, Germany). The cryosections (15 μm) were washed with warm phosphate buffered saline (PBS) solution and then incubated with 5 μM DHE for 30 min at 37°C in PBS. DHE specifically reacts with superoxide anions and converts to the red fluorescence compound ethidium (Figure 5). The sections were then examined and photographed by using an inverted fluorescent microscope equipped with a digital camera. Three sections (1.7 mm from Bregma) were measured from each brain and the mean of them was used for data analysis. Measurements were taken at 200 μm intervals on each image using image processing software as previously described (15). For these analyses, the experimenter was blinded to the treatment assignations. 

*Statistical analysis*

Data are expressed as mean ± SD. Infarct volumes were analyzed by one-way ANOVA and Laser Doppler data were compared by two-way ANOVA. Individual differences were determined by Tukey’s test. Differences of brain edema and ROS level between control and postconditioning (PC10) groups were analyzed by t-test. Nonparametric Mann-Whitney test was used for neurological deficits comparison. 

## Results

*Effect of postconditioning on blood flow of MCA territory*

**Figure 2:**
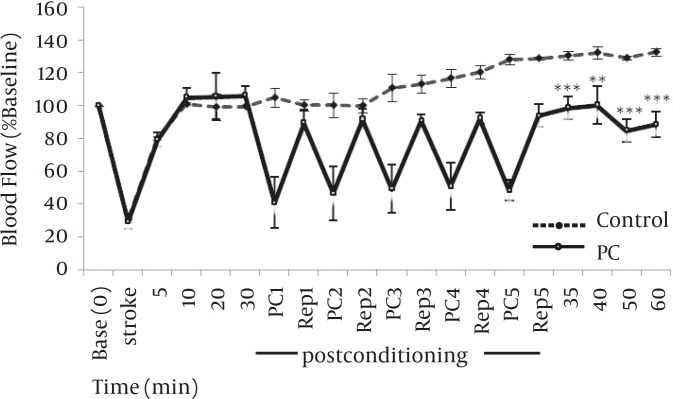
Effect of postconditioning (5 cycles of occluding and releasing common carotid arteries for 10 or 30 sec, respectively) at 30 min after stroke on cerebral blood flow (CBF). Blood flow decreased to approximately 70% of baseline (before stroke) immediately after stroke insult, returned to approximately 80 to 100% of baseline during 5 to 30 min after stroke in both control (n=5) and postconditioning (n=6) groups. Postconditioning hindered hyperemia of MCA territory at 35, 40, 50 and 60 min after embolic stroke. **P<0.005, ***P< 0.001

There were no significant differences in initial mean laser doppler readings between treatment groups. Percentage laser Doppler reductions were 72±6 and 73±5% for control and PC10 treatment groups, respectively. Compared to the control group, postconditioning with 10 sec occlusion and 30 sec reperfusion (PC10) prevented hyperperfusion at MCA territory when measured at 35, 40, 50 and 60 min after embolic stroke (*P*<0.005; Figure 2). 

*Effect of postconditioning on infarct volume and brain edema*

**Figure 3:**
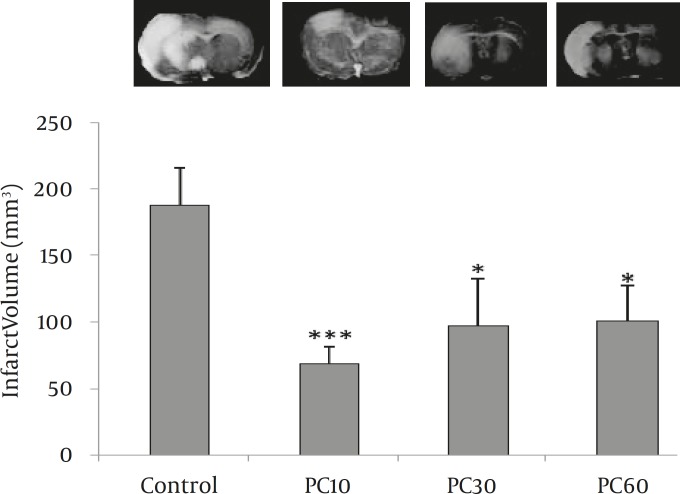
Effect of different types of postconditioning on infarct volume. Infarct volume was determined by calculating TTC stained brain sections that were obtained 48 hr after MCA (middle cerebral artery) embolization. The data are presented as Mean ± SD. In type 1 postconditioning (PC10), the CCAs occluding and releasing times were 10 and 30 sec, respectively and repeated for 5 cycles. In type 2 (PC30) and type 3 (PC60), both occluding and releasing times were 30 or 60 sec, respectively and repeated for 5 cycles. *P<0.05, ***P<0.001 compared to the control group

**Figure 4:**
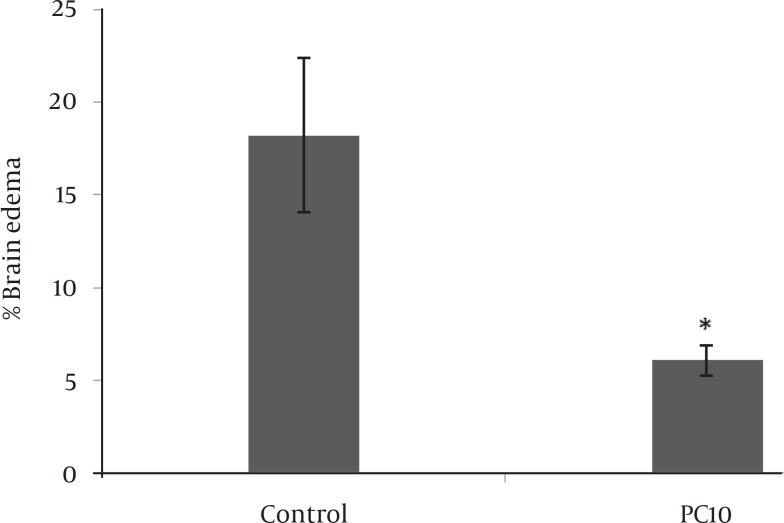
Effect of postconditioning on brain edema which was calculated by TTC stained brain sections obtained 48 hr after MCA (middle cerebral artery) embolization. The data are presented as Mean±SD PC10=postconditioning which was induced by 5 cycles of occluding and releasing CCAs with 10 and 30 sec, respectively. *P<0.05 compared to the control group

We did not observe any cerebral infarction and neurological deficits in sham-operated animals. Inducing postconditioning with 10 sec occlusion and 30 sec reperfusion (PC10) reduced infarct volume by approximately 64% compared to the control group (*P<*0.001). The rest postconditioning protocols also reduced infarct volume by approximately 45% compared to the control group (*P<*0.05; Figure 3) but they did not effect other parameters including brain edema or neurological deficits. Since better neuroprotection was observed by PC10, the other patterns of postconditioning were excluded from further study. Compared to the control group, PC10 decreased brain edema at 48 hr following the embolic stroke (*P<*0.05; Figure 4).

*Effect of postconditioning on ROS activity and neurologic functions*

**Figure 5:**
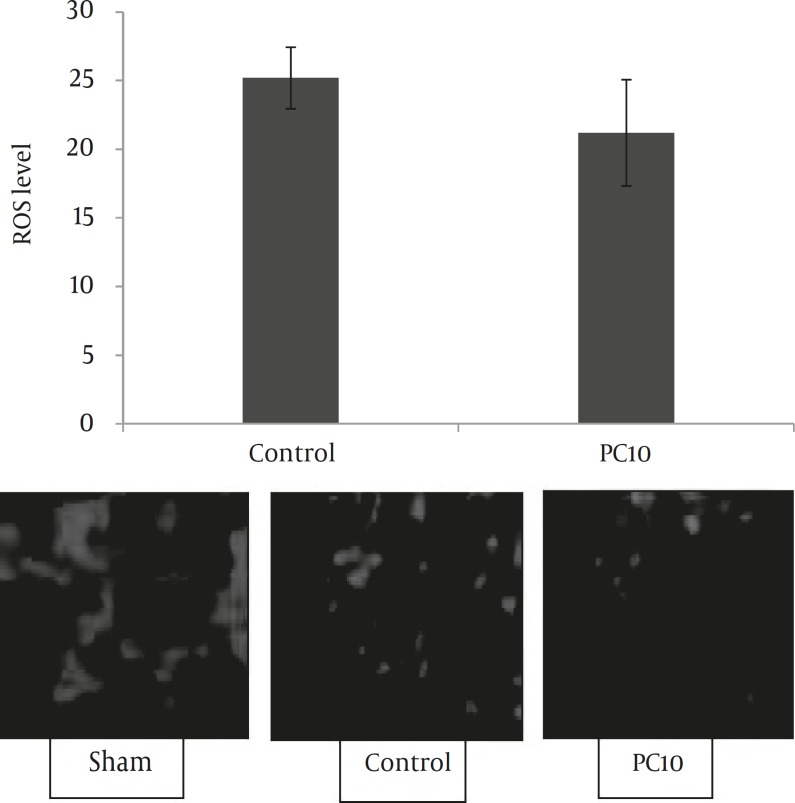
Effect of postconditioning on ROS levels after the embolic model of stroke which was calculated by dihydroethidium stained brain cryosections obtained 48 hr after MCA embolization. There is no significant difference between groups

We did not observe any significant change between PC10 and control group in ROS levels at 48 hr following the stroke (Figure 5). Postconditioning with 5 cycles of 10 sec occlusion and 30 sec reperfusion ameliorated neurological function at 48 hr following the embolic model of stroke (*P<*0.05). There was no significant difference in neurologic deficits between control and PC10 groups at 24 hr after MCA embolization (Table 1).

**Table 1:** Postconditioning decreased neurological deficits at 48 hr following the stroke

hr	Control	Postconditioning
24	3 (1.75-3.25)	3 (1.5-3)
48	3 (2-3.5)	2 (1-2)*

Neurological deficits were measured by a five-score scale at 24 and 48 hr following the embolic cerebral ischemia onset. Postconditioning was induced at 30 min after stroke. The data are presented as median, 25th and 75th percentiles (percentiles in the parentheses). Non parametric Mann-Whitney test showed a significant difference between control and postconditioning treated groups at 48 hr after embolic stroke. *P<0.05 compared to the control group at the same time

## Discussion

The main findings of this study are that postconditioning with 5 cycles of 10 sec occlusion and 30 sec reperfusion (PC10) of bilateral common carotid arteries reduces ischemic damage and neurological deficits induced by embolic stroke in rats. Many studies have demonstrated properties of postconditioning in permanent (4, 5, 16), transient (6) or global (17) cerebral ischemia. However, the reperfusion pattern of embolic stroke differs from the above mentioned models. To the best of our knowledge, there is no investigation studying effects of postconditioning on embolic stroke. This model of cerebral ischemia mimics human stroke and is more relevant to the pathophysiological situation in patients (18).

Our data revealed that postconditioning abolishes reactive hyperemia after cerebral ischemia insult. Reopening of the thrombotic occluded blood vessels is one the only strategy for acute stroke therapy by using recombinant tissue plasminogen activator (rt-PA) (19). However, abrupt reperfusion by rt-PA may cause an overproduction of reactive oxygen species, leading to reperfusion injury that exacerbates stroke (4, 5, 20). Reperfusion injury has been confirmed repeatedly in many studies (21). An overt hyper perfusion of cerebral ischemic tissue following similar conditions to those in the present study has been reported by others (5, 21). In our current embolic model of stroke and in a permanent MCA occlusion plus 15 min of bilateral CCA occlusion a hyperemia response occurred (5), which was disturbed by postconditioning. 

Although the effects of ischemic postconditioning have been confirmed, the mechanisms involved are poorly understood. It is well known that generation of excessive ROS during hyper reperfusion plays a major role in brain injury associated with cerebral ischemia (6). Based on other investigators (5, 22), prevention of reactive hyperemia may involve in effect of postconditioning after stroke through inhibition of ROS production. It has been reported that Toll-like receptor 4 signaling and innate immunity may be involved in the protective mechanisms of postconditioning and ischemic tolerance (23). Many investigations have shown that postconditioning inhibits apoptosis (6, 7, 22), ROS production (16) and activation of the protein kinase Akt after stroke (24). However, we did not observe any significant change in ROS level, following induction of postconditioning, which is not parallel with other recently findings. Since the pathophysiology of embolic stroke is different from the permanent or transient models (25), it may be elicited that perhaps there are different mechanisms for postconditioning in embolic stroke which needs further studies to be addressed.

As free radicals contribute to ischemic injury, many studies were done on effects of postconditioning on free radicals after stroke. Postconditioning robustly attenuated the amount of superoxide at 30 min after reperfusion in the model of 30 min of CCA occlusion plus permanent MCA occlusion (5, 16, 20). The result of one study is against our findings and other investigators which showed postconditioning might not influence the early brain damage induced by focal cerebral ischemia in rats (8). In that study however, postconditioning was different from our current experiments as well as the others (6, 8, 13) as it was elicited by only 3 cycles of 30 sec reperfusion interspersed by 10 sec ischemia immediately after onset of reperfusion. Again the infarct ratios, brain edema ratios and motor behavior deficits were analyzed 24 hr after ischemic insult (8), while longer time points are needed for evaluation of behavioral deficits and infarction. 

Our study has some limitations. The effects of therapeutic time window of ischemic postconditioning on embolic model of stroke have not been investigated in the current study. However, it has been reported that delayed postconditioning robustly reduced the infarct size, and improved the outcomes of behavioral tests up to two months after transient model of stroke (4). In the current study, the effect of postconditioning on embolic stroke without application of thrombolytic therapy was investigated, while reperfusion in ischemic stroke patients is usually achieved by r-tPA. It has been reported that delayed postconditioning in the transient model of stroke counteracts the exacerbating effect of the r-tPA, and found that delayed postconditioning mitigated the worsening effect of the t-PA on infarction (4, 21). However, in this study, reperfusion was induced by bilateral CCA release. Further studies are required to test whether postconditioning shows effects in an embolic model, in which reperfusion is induced by r-tPA application.

## Conclusion

The present study indicates for the first time, that postconditioning with 5 cycles of 10 sec occlusion and 30 sec reperfusion of bilateral common carotid arteries, applied at 30 min after embolic stroke, decreases hyperemia after injury insult, reduces infarct volume and brain edema, and improves neurological functions. However, further investigations are required to elucidate the exact effects of postconditioning on embolic stroke after achieving reperfusion by r-tPA. 
